# Induction chemotherapy with paclitaxel, carboplatin and cetuximab for locoregionally advanced nasopharyngeal carcinoma: A single-center, retrospective study

**DOI:** 10.3389/fonc.2022.951387

**Published:** 2022-08-11

**Authors:** Naohiro Takeshita, Tomohiro Enokida, Susumu Okano, Takao Fujisawa, Akihisa Wada, Masanobu Sato, Hideki Tanaka, Nobukazu Tanaka, Atsushi Motegi, Sadamoto Zenda, Tetsuo Akimoto, Makoto Tahara

**Affiliations:** ^1^ Department of Head and Neck Medical Oncology, National Cancer Center Hospital East, Kashiwa, Japan; ^2^ Department of Radiation Oncology, National Cancer Center Hospital East, Kashiwa, Japan

**Keywords:** LA-NPC, induction chemotherapy, PCE, cetuximab, chemoradiotherapy

## Abstract

**Background:**

The addition of induction chemotherapy (IC) before chemoradiotherapy (CRT) has improved survival over CRT alone in locoregionally advanced nasopharyngeal cancer (LA-NPC). Nevertheless, this population would benefit from further development of a novel IC regimen with satisfactory efficacy and a more favorable safety profile.

**Methods:**

We retrospectively assessed 29 LA-NPC patients who received the combination of paclitaxel (PTX), carboplatin (CBDCA), and cetuximab (Cmab) (PCE) as IC (IC-PCE) at the National Cancer Center Hospital East between March 2017 and April 2021. IC-PCE consisted of CBDCA area under the plasma concentration-time curve (AUC) = 1.5, PTX 80 mg/m^2^, and Cmab with an initial dose of 400 mg/m^2^ followed by 250 mg/m^2^ administered weekly for a maximum of eight weeks.

**Results:**

Patient characteristics were as follows: median age, 59 years (range 24–75); 0, 1 performance status (PS), 25, 4 patients; and clinical stage III/IVA/IVB, 6/10/13. The median number of PCE cycles was 8(1-8). After IC-PCE, 26 patients received concurrent cisplatin and radiotherapy (CDDP-RT), one received concurrent carboplatin/5-fluorouracil and radiotherapy (CBDCA/5-FU-RT), and two received RT alone. The % completion of CDDP-RT was 88.5%. The response rate was 75.9% by IC and 100% at completion of CRT. The 3-year recurrence-free survival, locoregional failure-free survival, distant recurrence-free survival, and overall survival were 75.9%, 79.3%, 84.3%, and 96.3%, respectively. The incidence of adverse events of grade 3/4 was 34.5% during IC and 44.8% during CRT.

**Conclusion:**

IC-PCE is feasible and effective for LA-NPC and may be a treatment option for this disease.

## Introduction

Worldwide, nasopharyngeal carcinoma (NPC) affected 133,354 patients and caused 80,008 deaths in 2020 (1). Prevalence is high in South China, Southeastern Asia, and North Africa. More than 70% of patients are diagnosed with locally advanced disease at presentation (2). Because of the anatomical location and high sensitivity of NPC to radiotherapy (RT) and chemotherapy, chemoradiotherapy (CRT) is the backbone of treatment. Moreover, the addition of chemotherapy as induction chemotherapy (IC) or adjuvant chemotherapy to CRT is now a standard treatment for this disease (3). Surprisingly, several prospective studies have shown that IC consistently results in higher response and exerts a pronounced effect on survival and distant metastasis (4–7). A meta-analysis also showed that the addition of IC to CRT improved progression-free survival (PFS) and overall survival (OS) (8–10). Based on these findings, platinum-based IC, herein the combination of docetaxel (DTX), cisplatin (CDDP), and fluorouracil (TPF) and gemcitabine (GEM)+CDDP (GP) followed by CRT, are now considered standard therapy over CRT alone for this patient population (4, 5).

However, GEM has not been approved for the treatment of NPC in several countries, including Japan, which hampers use of the drug as a part of IC. Further, IC-TPF as a CDDP-containing triplet regimen sometimes raises concerns about treatment-related toxicity, such as renal impairment related to repeated administration of CDDP over time and increased myelosuppression. Moreover, despite several studies suggesting that compliance with cisplatin during CRT is a critical factor in maximizing its efficacy (11–13), compliance with concomitant chemotherapy with RT following IC is generally impaired due to the adverse effects of the prior IC (9).

We previously tested the combination of paclitaxel (PTX), carboplatin (CBDCA), and cetuximab (Cmab) (PCE) as IC in a Japanese multicenter phase II trial in patients with unresectable locally advanced head and neck squamous cell carcinoma (LA-HNSCC) arising from the hypopharynx, oropharynx, and larynx (14). Results showed that PCE as IC(IC-PCE) was feasible and effective, with a response rate of 88.6% by IC, and had no effect on compliance with subsequent CRT with CDDP. Further, PCE has shown promising efficacy in recurrent or metastatic NPC (R/M NPC), represented by an overall response rate (ORR) of 58.3% (15).

Here, we investigated whether IC-PCE would also be an effective treatment option with a favorable toxicity profile for LA-NPC.

## Materials and methods

### Patient selection

We retrospectively reviewed LA-NPC patients treated with IC-PCE from March 2017 to April 2021 at the National Cancer Center Hospital East, Japan. Inclusion criteria were as follows (1): pathologically proven NPC, (2) newly diagnosed non-distant metastatic stage III to IVB disease (except T3–4N0; 7th Union for International Cancer Control and American Joint Committee on Cancer), and (3) no other active malignant tumor during treatment. This study was approved by the Institutional Review Board of the National Cancer Center Hospital East.

### Treatment

The induction PCE regimen consisted of CBDCA area under the plasma concentration-time curve (AUC) = 1.5, PTX 80 mg/m^2^, and Cmab with an initial dose of 400 mg/m^2^ followed by 250 mg/m^2^ administered weekly for eight weeks. Following IC, concurrent chemoradiotherapy was started. During CRT, cisplatin was administered intravenously at a dose of 80 mg/m^2^ every three weeks on days 1, 22, and 43; or at a dose of 20 mg/m^2^ on days 1-4, repeated three times at 3-week intervals. For one patient who received chemoradiotherapy consisting of RT plus the combination of CBDCA and 5-fluorouracil (5-FU), CBDCA (AUC = 5 on day 1) and 5-FU (500mg/m^2^ by 24-h continuous infusion on days 1-4) was administered every four weeks for up to two cycles. As with RT, all patients were treated with intensity-modulated radiation therapy (IMRT). The planned total radiation dose was 70 Gy in 35 fractions (2 Gy per day) or 69.96 Gy in 33 fractions (2.12 Gy per day), with prophylactic dose (56 - 63 Gy) irradiation to the elective neck. Toxicity during treatment was graded using the Common Toxicity Criteria for Adverse Events (CTCAE version 4.0).

### Evaluation of efficacy and statistical analysis

Clinical tumor response to treatment was evaluated radiographically according to Response Evaluation Criteria in Solid Tumors (RECIST) ver. 1.1 using computerized tomography (CT) or magnetic resonance imaging (MRI) and [18F]-fluorodeoxyglucose positron-emission tomography (PET)/CT fusion imaging, as required. OS, Recurrence-free survival (RFS), locoregional failure-free survival (LFFS) and distant recurrence-free survival (DRFS) were calculated by the Kaplan–Meier product-limit method. OS was defined as the period from the first day of IC-PCE until death from any cause. RFS was calculated from the first day of IC-PCE until disease recurrence, disease progression or death from any cause. LFFS was calculated from the first day of IC-PCE until recurrence in the primary tumor or a local/regional lymph node, or death from any cause. DRFS was calculated from the first day of IC-PCE until distant metastasis, or death from any cause. For patients who were treated with CDDP plus RT following IC-PCE, the proportion of CRT completion (%CRT completion) was defined by (a) completion of cumulative CDDP dose ≥ 200 mg/m^2^; and (b) completion of radiotherapy within two weeks after the planned completion date. Patients who were lost to follow-up or remained alive without a specified event were censored at the date of the last follow-up. Differences in LFFS by compliance with CDDP during CRT, differences in DRFS by the number of IC-PCE cycles, and differences in RFS by the number of IC-PCE cycles and compliance with CDDP during CRT were assessed using stratified log-rank tests. Hazard ratios (HRs) were calculated by Cox regression analysis. All statistical analyses were performed with EZR (version.1.51; Saitama Medical Center, Jichi Medical University, Saitama, Japan), which is a graphical user interface for R (The R Foundation for Statistical Computing, Vienna, Austria; version.4.1.1). More precisely, it is a modified version of R commander which is designed to add statistical functions frequently used in biostatistics (16).

## Results

### Patients characteristics

During the study period, 29 LA-NPC patients were included in the study. Baseline patient characteristics are presented in **Table 1**. Median age was 59 years (24−75 years), and all patients had an ECOG performance status (PS) of 0 to 1. Of the 29 patients, 22 (75.9%) were males, and 6 (20.7%), 10 (34.5%), and 13 (44.8%) were diagnosed with stage III, IVA and IVB disease, respectively (**Supplementary Figure 1**).

**Table 1:** Patient characteristics (N=29).

Characteristic	No. of patients (%)
**Age** [years]	
Median (range)	59 (24-75)
**Gender**	
Male/female	22 (75.9)/7 (24.1)
**ECOG PS**	
0/1	25 (86.2)/4 (13.8)
**T category**	
1	7 (24.1)
2	2 (6.9)
3	4 (13.8)
4	16 (55.2)
**N category**	
1	8 (27.6)
2	8 (27.6)
3a	0 (0)
3b	13 (44.8)
**M category**	
0	29 (100)
1	0 (0)
**Stage** ^†^	
III	6 (20.7)
IVA	10 (34.5)
IVB	13 (44.8)
**Histology**	
Non-keratinizing carcinoma differentiated subtype	8 (27.6)
Non-keratinizing carcinoma undifferentiated subtype	18 (62.1)
Squamous cell carcinoma	3 (10.3)
**EBV status**	
Positive	18 (62.1)
Negative	1 (3.4)
Unknow	10 (34.5)
**Smoking status**	
Never	12 (41.4)
Former	13 (44.8)
Current	4 (13.8)
**Cigarette smoker** ^‡^	
<10	16 (55.1)
≥10	13 (44.8)

ECOG PS, Eastern Cooperative Oncology Group Performance Status; EBV, Epstein-Barr virus; SD, standard deviation. ^†^AJCC 7th. ^‡^ Among former or current smokers.

### Treatment delivery and antitumor efficacy

**Figure 1:**
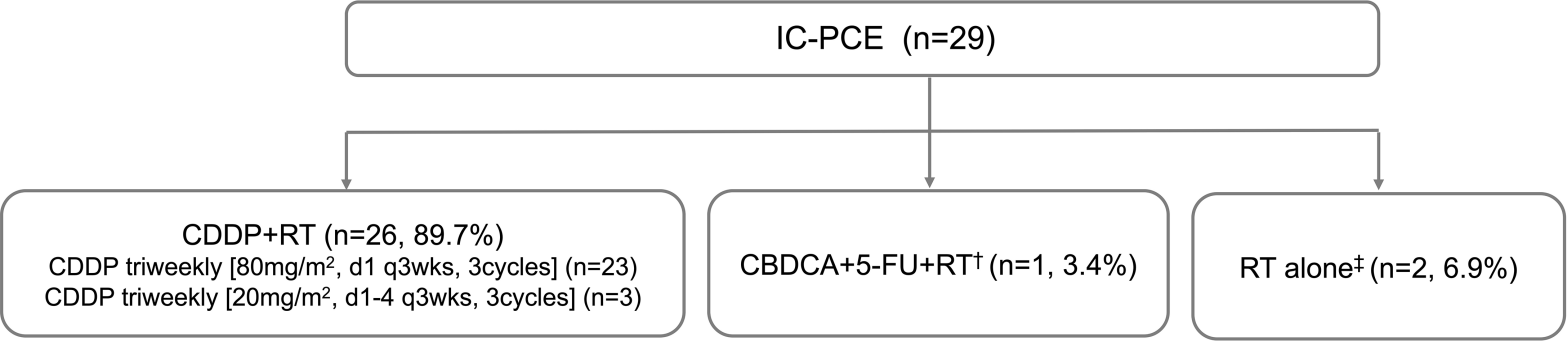
Patient flow diagram of treatment delivery. IC, induction chemotherapy; CDDP, cisplatin; RT, radiotherapy; CBDCA, carboplatin; FU, fluorouracil. ^†^Ineligible for CDDP because of decreased ejection fraction. ^‡^Originally planned to receive CDDP+RT but CDDP could not be administered due to tumor infection or pneumonia.

**Figure 2:**
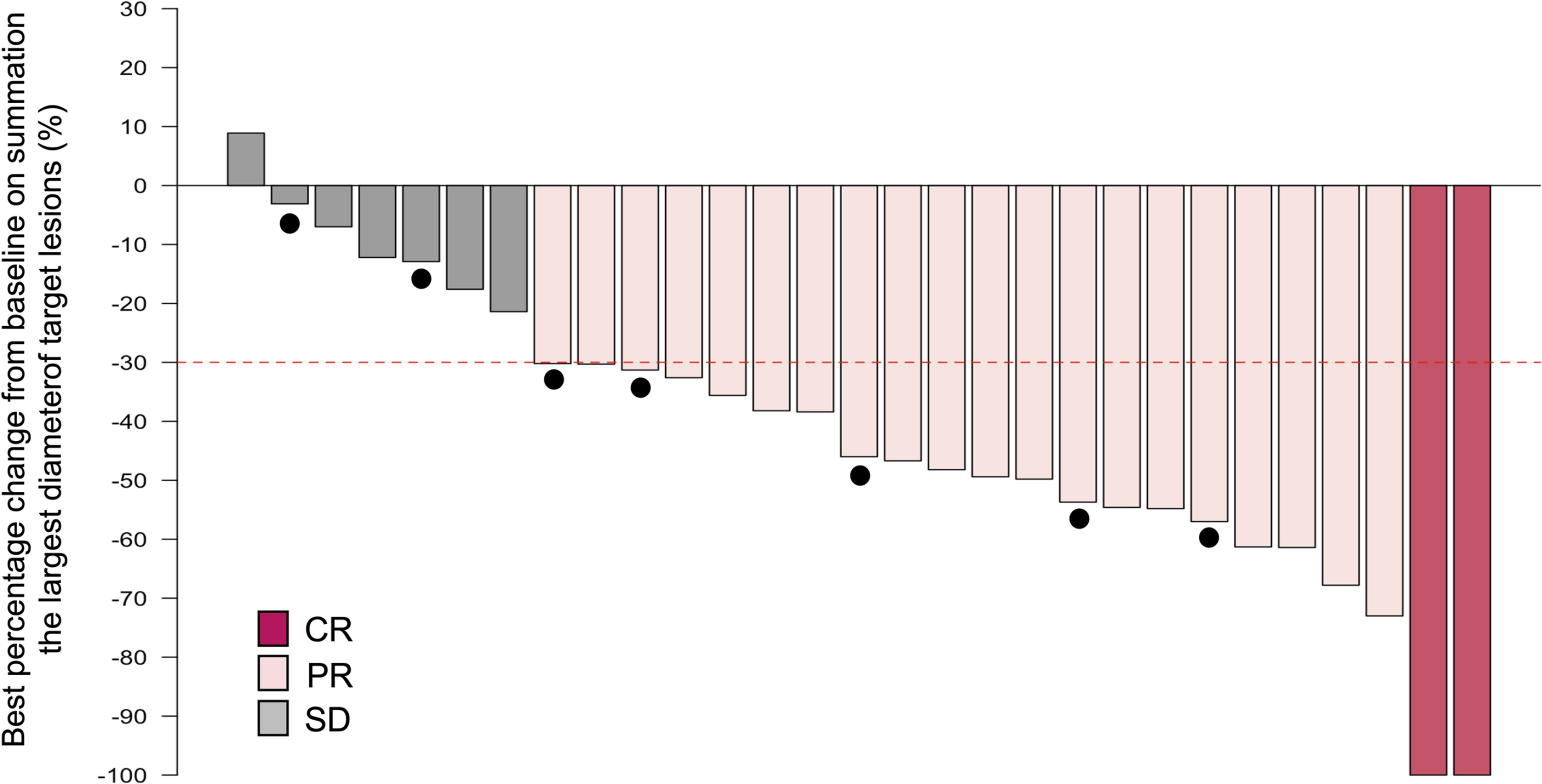
Waterfall plot of the maximum percentage change from baseline on summation of the largest diameter of target lesions by induction chemotherapy. Note that the dashed line indicates a 30% reduction in tumor burden in the target lesion and closed circles indicate cases with late recurrence. CI, confidence interval; CR, complete response; PR, partial response; SD, stable disease; PD, progressive disease.

In this setting, in which the maximum number of PCE cycles is eight, the median number of administered PCE cycles reached 8 (range, 1-8). A total of 16 patients (55.2%) completed the eight cycles of planned IC. Reasons for discontinuation are described in **Supplementary Table 1**. Median time from the first visit to starting PCE was 11 days (range, 1-32). Among 29 patients receiving IC-PCE, 26 (89.7%) patients underwent CDDP+RT (**Figure 1**). One patient received CBDCA+5-FU+RT because of a decreased cardiac ejection fraction before initiating IC-PCE. Two patients received RT alone following IC-PCE, one with tumor infection and the second with repeated aspiration pneumonia after completion of IC. The objective response rate (ORR) after IC-PCE was 75.9%, including two patients (6.9%) with complete response (CR) and 20 (69.0%) with partial response (PR) (**Table 2**). Only one (3.4%) of 29 patients experienced tumor growth in size in the IC phase (**Figure 2**). When we focused on local therapy in the 26 patients who received CDDP+RT, RT with 70 Gy in 35 fractions and 69.96 Gy in 33 fractions was delivered to 10 and 16 patients, respectively. None required the temporary cessation of RT, resulting in a median length of RT of 48 days. %CRT completion in these 26 patients was 88.5% [95% confidence interval (CI), 70.2% - 96.8%] (**Supplementary Table 3**). After completing CRT or RT, ORR reached 100%, with CR in 28 patients (96.6%) and PR in one patient (3.4%) (**Table 2**).

**Table 2:** Response to treatment by RECIST ver.1.1.

	CR (%)	PR (%)	SD (%)	PD (%)	%RR (95%CI)
**Induction chemotherapy**	2 (6.9)	20 (69.0)	7 (24.1)	0 (0)	75.9% (57.6-88.0)
**Chemoradiotherapy/** **radiotherapy**	28 (96.6)	1 (3.4)	0 (0)	0 (0)	100% (86.1-102.2)

%RR, proportion of CR+PR. RECIST, Response Evaluation Criteria in Solid Tumors; CI, confidence interval; CR, complete response; PR, partial response; SD, stable disease; PD, progressive disease.

### Treatment outcome

**Figure 3:**
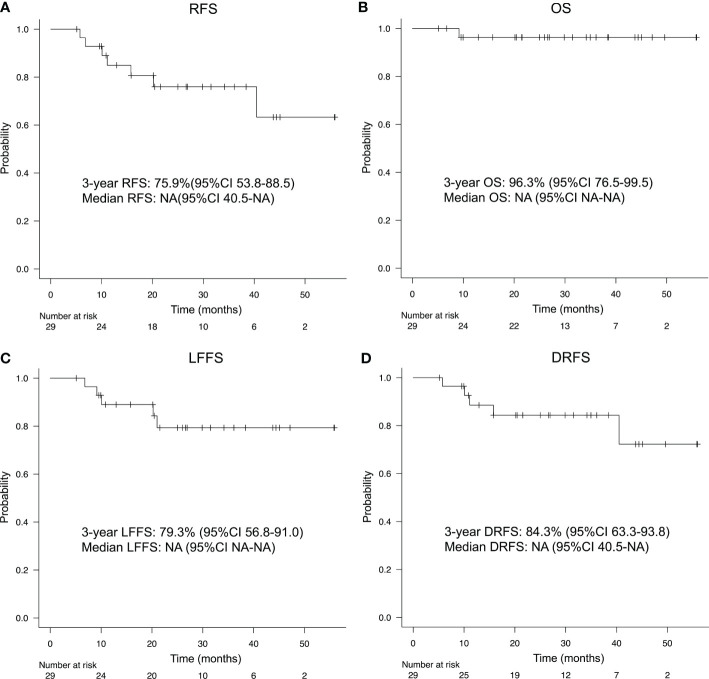
**(A)** Recurrence-free survival (RFS), **(B)** overall survival (OS), **(C)** locoregional failure-free survival (LFFS), and **(D)** distant recurrence-free survival (DRFS) of patients treated with IC-PCE. IC, induction chemotherapy; CI, confidence interval; NA, not available.

Median follow-up time was 26.9 months (range, 5.1–56.0 months). Details regarding patterns of relapse and subsequent therapies after recurrence are provided in **Supplementary Figure 2**. The 3-year RFS and OS were 75.9% (95% CI, 53.8-88.5) and 96.3% (95%CI, 76.5-99.5), respectively (**Figures 3A**, **B**). The 3-year LFFS and DRFS were 79.3% (95% CI, 56.8-91.0) and 84.3% (95%CI, 63.3-93.8), respectively (**Figures 3C**, **D**). In addition, we also observed a trend toward improved DRFS in patients with a higher number of IC-PCE cycles and a statistically significantly favorable LFFS in patients with the completion of cumulative CDDP dose ≥ 200mg/m^2^ (**Supplementary Figures 3A**, **B**). Altogether, there were trends for improvement in RFS in patients with the completion of IC-PCE 8 cycles and the completion of cumulative CDDP dose ≥ 200mg/m^2^ (**Supplementary Figure 3C**). For the 26 patients who received CDDP+RT following IC-PCE, the 3-year RFS, OS, LFFS and DRFS were 81.7% (95% CI, 57.8-92.8), 95.8% (95%CI, 73.9-99.4), 86.6% (95% CI, 63.4-95.5) and 91.2% (95%CI, 69.0-97.7), respectively (**Supplementary Figure 4**). Furthermore, four patients developed local recurrence, three patients had local recurrence within the high-dose zone (cumulative RT dose: 70Gy), and the remaining patient developed recurrence within the prophylactic dose zone (cumulative RT dose: 63Gy) of their IMRT. While from the viewpoint of the use of the anti-tumor drug during RT, two patients who developed local recurrence were treated with RT alone without concurrent chemotherapy.

### Adverse events

Acute toxicities experienced during IC-PCE and CRT are listed in **Tables 3**, **4**, respectively. Common grade 3/4 adverse events during IC-PCE were neutropenia (24.1%), leukopenia (10.4%), and skin rash (6.9%). In contrast, mucositis (20.7%), leukopenia (17.2%), and neutropenia (17.2%) were the most frequently observed grade 3/4 adverse events during CRT. The total frequency of grade 3/4 toxicity in the IC and CRT phases was 34.5% and 44.8%, respectively. There was no treatment-related death throughout treatment.

**Table 3:** Selected toxicity during induction chemotherapy.

		No. of patients[n=29] (%)			
		Grade			
	All grade	1	2	3	4
**Hematological toxicity**					
Leukopenia	17 (58.6)	13 (44.8)	1 (3.4)	3 (10.3)	0 (0)
Neutropenia	17 (58.6)	6 (20.7)	4 (13.8)	6 (20.7)	1 (3.4)
Febrile neutropenia	1 (3.4)	0 (0)	0 (0)	1 (3.4)	0 (0)
Anemia	20 (69.0)	19 (65.5)	1 (3.4)	0 (0)	0 (0)
Thrombocytopenia	3 (10.3)	2 (6.9)	1 (3.4)	0 (0)	0 (0)
**Non-hematological toxicity**					
Infusion reaction	0 (0)	0 (0)	0 (0)	0 (0)	0 (0)
AST elevation	16 (55.2)	13 (44.8)	3 (10.3)	0 (0)	0 (0)
ALT elevation	18 (62.1)	15 (51.7)	2 (6.9)	1 (3.4)	0 (0)
Creatinine increased	2 (6.9)	0 (0)	2 (6.9)	0 (0)	0 (0)
Nausea	7 (24.1)	6 (20.7)	1 (3.4)	0 (0)	0 (0)
Dysgeusia	0 (0)	0 (0)	0 (0)	0 (0)	0 (0)
Mucositis	7 (24.1)	5 (17.2)	2 (6.9)	0 (0)	0 (0)
Fatigue	5 (17.2)	5 (17.2)	0 (0)	0 (0)	0 (0)
Peripheral neuropathy	9 (31.0)	7 (24.1)	2 (6.9)	0 (0)	0 (0)
Alopecia	8 (27.6)	7 (24.1)	1 (3.4)	0 (0)	0 (0)
Rash	25 (86.2)	13 (44.8)	10 (34.5)	2 (6.9)	0 (0)
Other skin^†^	4 (13.8)	4 (13.8)	0 (0)	0 (0)	0 (0)
Respiratory disorders^††^	1 (3.4)	0 (0)	1 (3.4)	0 (0)	0 (0)
Thromboembolic event	1 (3.4)	0 (0)	1 (3.4)	0 (0)	0 (0)
Pneumonitis	0 (0)	0 (0)	0 (0)	0 (0)	0 (0)
Soft tissue infection	2 (6.9)	0 (0)	2 (6.9)	0 (0)	0 (0)
**Total with ≥Grade 3 toxicity**	10 (34.5)				

Graded according to common toxicity criteria for adverse events version 4.0. ALT, alanine aminotransferase; AST, aspartate amino transferase. ^†^Including seborrheic dermatitis, dry skin, pruritus and skin cracks. ^††^Acute exacerbation of chronic obstructive pulmonary disease.

**Table 4:** Selected toxicity during chemoradiotherapy.

		No. of patients[n=29] (%)			
		Grade			
	All grade	1	2	3	4
**Hematological toxicity**					
Leukopenia	26 (89.7)	14 (48.2)	7 (24.1)	5 (17.2)	0 (0)
Neutropenia	26 (89.7)	10 (34.5)	11 (38.0)	5 (17.2)	0 (0)
Febrile neutropenia	1 (3.4)	0 (0)	0 (0)	1 (3.4)	0 (0)
Anemia	28 (96.6)	25 (86.2)	3 (10.3)	0 (0)	0 (0)
Thrombocytopenia	7 (24.1)	4 (13.8)	3 (10.3)	0 (0)	0 (0)
**Non-hematological toxicity**					
AST elevation	11 (28.0)	11 (38.0)	0 (0)	0 (0)	0 (0)
ALT elevation	16 (55.2)	15 (51.7)	1 (3.4)	0 (0)	0 (0)
Creatinine increased	4 (13.8)	4 (13.8)	0 (0)	0 (0)	0 (0)
Nausea	17 (58.6)	10 (34.5)	6 (20.7)	1 (3.4)	0 (0)
Dysgeusia	19 (65.5)	8 (27.6)	11 (38.0)	0 (0)	0 (0)
Mucositis	24 (82.8)	20 (70.0)	4 (13.8)	6 (20.7)	0 (0)
Dry mouth	24 (82.8)	20 (70.0)	4 (13.8)	0 (0)	0 (0)
Fatigue	22 (75.9)	9 (31.0)	13 (44.8)	0 (0)	0 (0)
Constipation	8 (27.6)	6 (20.7)	2 (6.9)	0 (0)	0 (0)
Radiation dermatitis	29 (100)	20 (70.0)	9 (31.0)	0 (0)	0 (0)
**Total with ≥Grade 3 toxicity**	13 (44.8)				

Graded according to common toxicity criteria for adverse events version 4.0. ALT, alanine aminotransferase; AST, aspartate aminotransferase.

## Discussion

This is the first study to indicate the feasibility and efficacy of PCE as IC for far-advanced LA-NPC. After completing locoregional therapy following IC-PCE, the ORR was 100%, and 3-year OS reached 96.3%.

Following the initial randomized phase II trial which found that the addition of two cycles of DTX and CDDP as induction therapy prior to CRT significantly improved 3-year OS (94% *vs*. 68%, HR, 0.24; 95% CI, 0.08 to 0.73) (6), reproduced evidence had shown that IC followed by CRT with cisplatin improves survival outcomes over CRT alone for LA-NPC (4, 5), with a positive effect primarily on distant control. However, these cisplatin-containing IC regimens can produce unacceptable toxicities such as severe renal impairment as well as myelosuppression which may cause treatment-related death, and sometimes compromise compliance with locoregional therapy following induction therapy, especially in daily practice. Thus, further development of a novel IC regimen with satisfactory efficacy and favorable safety profile has been warranted.

Given the frequency of epidermal growth factor receptor (EGFR) expression on NPC, in 73.3-84.1% of cases (17–20), several studies evaluated treatment with anti-EGFR therapy in collaboration with CRT for LA-NPC (21–23) and with chemotherapy for R/M NPC (15, 24–26), and reported promising clinical efficacy against these diseases. As an example of the former, Hao et al. revealed that adding the anti-EGFR monoclonal antibodies Cmab/nimotuzumab (NTZ) to IC was statistically significantly associated with improved OS compared with IC without these anti-EGFR drugs (IC *vs*. IC + Cmab/NTZ, HR,1.984; 95% CI, 1.023 to 3.848) (27). Ying et al. also showed that NTZ combined with cisplatin plus 5-fluorouracil as IC had a better lymph node response rate with milder adverse reactions than those in the TPF regimen (28). These suggest that Cmab-containing regimens may be worth testing in anticipation of an improved prognosis. Indeed, we recently reported that the PCE regimen was effective even in patients with R/M NPC (n=14), among whom 92% of evaluable patients experienced tumor shrinkage, with ORR and CR rates of 58.3% and 16.7%, respectively (15). However, no study has evaluated IC-PCE in the treatment of LA-NPC.

In the present study, anti-tumor efficacy and prognosis were equivalent or limited to slightly inferior to those in previous reports using IC-TPF (4) and -GP (5). This is notwithstanding the apparently far-advanced disease state in our cohort compared with these previous studies (eg. N-stage 3b: current study 44.8%, IC-TPF 11%, and IC-GP 6.2%; and cStage IVB: current study 44.8%, IC-TPF 16%, and IC-GP 11.2%), as shown in **Supplementary Table 4**. Further, as we mention above, a favorable toxicity profile during the IC phase is a key factor not only for patient safety but also for maximizing treatment efficacy by maintaining local therapy following IC. From this perspective, IC-PCE appears to offer several advantages over other IC-regimens: the rate of grade 3/4 adverse events during IC-PCE was low, at 34.5%, compared to another IC-regimen for LA-NPC at 42.3% in the TPF study (4) and 38.9% in the GP study (5), even though our present study included more patients aged over 60 years [52% in the current study *vs*. 0% in the TPF study (4)]. These safety data are similar to the contrasting results observed among the previous two prospective studies of IC-PCE (14) and IC-TPF (29) in unresectable LA-SCCHN, in which grade 3/4 toxicity was less frequent in the former study (14.2% *vs*. 79%). We believe that IC-PCE’s more feasible toxicity profile contributed to better compliance with CRT following IC compared with other IC regimens. Supporting these presumptions, our present study also provided an equivalent or better prognosis and relatively safer toxicity profile than a previous Japanese retrospective study for LA-NPC; patients in that study had similar clinical characteristics and were treated with IC-TPF followed CDDP plus RT (1y-PFS:73%, 1y-OS: 91%, G3/4AE during IC-TPF: 73%) (30). Together, these studies suggest the promise of IC-PCE in terms of both efficacy and safety in managing this population.

We also obtained several suggestive findings from the present study regarding the potential meaning of IC and subsequent local therapy in controlling the disease in NPC. A meta-analysis showed a significant difference in completing all concomitant chemotherapy cycles in the CRT alone group compared to the IC group (9).

Specifically, a cumulative CDDP dose of 200 mg/m^2^ was enough to achieve significantly better locoregional control and survival than those without this dose (11–13). Based on this result, we initially set a target CDDP dose at CRT following IC of 240 mg/m^2^ (80 mg/m^2^, 3 cycles) as a reasonable and well-balanced threshold with regard to both efficacy and safety. Indeed, our present completion rate with a cumulative CDDP dose ≥ 200 mg/m^2^ during CRT reached 88.5%, which is closely similar to our previous phase II study (14); there was in fact a statistically significant difference in LFFS by compliance with CDDP during CRT (**Supplementary Figure 3**). We believe our initial attempt meets the purpose of treatment; however, at the same time, we expect that a higher dose (i.e., targeting 300mg/m^2^) of CDDP during CRT following IC-PCE can be given safely because of its favorable safety profile in the IC phase, and that it may further contribute to an improvement in patient prognosis, especially in patients with far-advanced disease. The point should be addressed in a prospective study with a larger patient cohort. Moreover, our study indicated that favorable compliance with IC was correlated with a reduced risk of distant failure (**Supplementary Figure 3**). This finding is also in accord with another meta-analysis showing a statistically significant 37% reduction in the hazard of developing distant metastases in favor of IC (8).

IC-PCE has several further advantages in addition to those described above. First, the regimen is safely delivered on an outpatient basis without such troublesome procedures as intravenous hydration, and can therefore be started immediately if needed. In this study, the median time from the first visit to starting PCE was as short as 11 days, with a range of 1-32. Considering that LA-NPC patients often experience disease-related symptoms, including severe pain as well as neurological manifestations, this ability to start treatment promptly can be of very substantial benefit to them. Second, thanks to its lower toxicity, the regimen can be used as a treatment option for fragile patients, including subjects who are ineligible for a cisplatin-containing IC regimen. Shirasu et al. demonstrated that 75% (18/24) of these patients completed IC-PCE with an ORR of 87% and equivalent incidence and severity of adverse events to that observed in studies for a cisplatin-eligible population with unresectable disease (31). Similarly, Rebecca et al. revealed that 86% (19/22) of frail or elderly patients with HNSCC successfully reached their endpoint with IC-PCE with Modified RECIST response rates (MRRR) of 64% (32). However, detailed exploration of the treatment flow diagram in the current study revealed two patients considered ineligible for CRT with CDDP due to infectious episodes in the IC phase; these patients were treated with RT alone, but both experienced late local recurrence (**Supplementary Figure 2**). Given that several alternative agents can be substituted for CDDP in CRT, such as carboplatin (33) and nedaplatin (34), which generally less frequently induce leucopenia/neutropenia - considered helpful in reducing the risk of infection - but which offer equivalent or non-inferior potency as RT sensitizers compared with CDDP, these drugs may be available when attempting to overcome treatment failure to achieve better local control. In contrast, for patients eligible for CDDP, adding Cmab to the CRT (herein CDDP + RT) may also be worth future evaluation, especially in IC-PCE responders, in whom Cmab can be a key drug (20, 23). By these means, the high feasibility and efficacy of IC-PCE should enable us to structure the most appropriate individualized treatment for each patient.

As a limitation, the present study was conducted under a retrospective design with a small number of patients at a single center, reducing its statistical power, particularly in the subgroup analyses. Indeed, although IC-PCE followed by locoregional therapy represented by CDDP plus RT is considered a promising treatment option for LA-NPC patients, no prospective study has yet compared this regimen with CRT alone, and clarifying its role in this field *via* a prospective randomized controlled trial with a larger sample size is therefore warranted. Accordingly, we are planning to conduct a study for both CDDP-eligible and -ineligible patients, and to comprehensively answer the questions raised but not concluded in the current study.

In conclusion, we demonstrated that IC-PCE is feasible and has promising efficacy in the treatment of far-advanced LA-NPC. In particular, 3-year RFS was 75.9% and 3-year OS was 96.3%. IC-PCE may be a suitable treatment option as IC for this population.

## Data availability statement

The raw data supporting the conclusions of this article will be made available by the authors, without undue reservation.

## Ethics statement

This study was reviewed and approved by Institutional Review Board of the National Cancer Center Hospital East. Written informed consent for participation was not required for this study in accordance with the national legislation and the institutional requirements.

## Author contributions

NaT, TE and MT participated in the study concept and design, interpreted the data, and drafted the manuscript. All authors contributed to the article and approved the submitted version.

## Conflict of interest

TE, SO and MT receive honoraria from Merck Biopharma.

The remaining authors declare that the research was conducted in the absence of any commercial or financial relationships that could be construed as a potential conflict of interest.

## Publisher’s note

All claims expressed in this article are solely those of the authors and do not necessarily represent those of their affiliated organizations, or those of the publisher, the editors and the reviewers. Any product that may be evaluated in this article, or claim that may be made by its manufacturer, is not guaranteed or endorsed by the publisher.
