# Supplementary figures and images for: Induction chemotherapy with paclitaxel, carboplatin and cetuximab for locoregionally advanced nasopharyngeal carcinoma: A single-center, retrospective study
Source: Front Oncol. 2022 Aug 11;12:951387. doi: 10.3389/fonc.2022.951387 (PMC9402945; doi:10.3389/fonc.2022.951387)

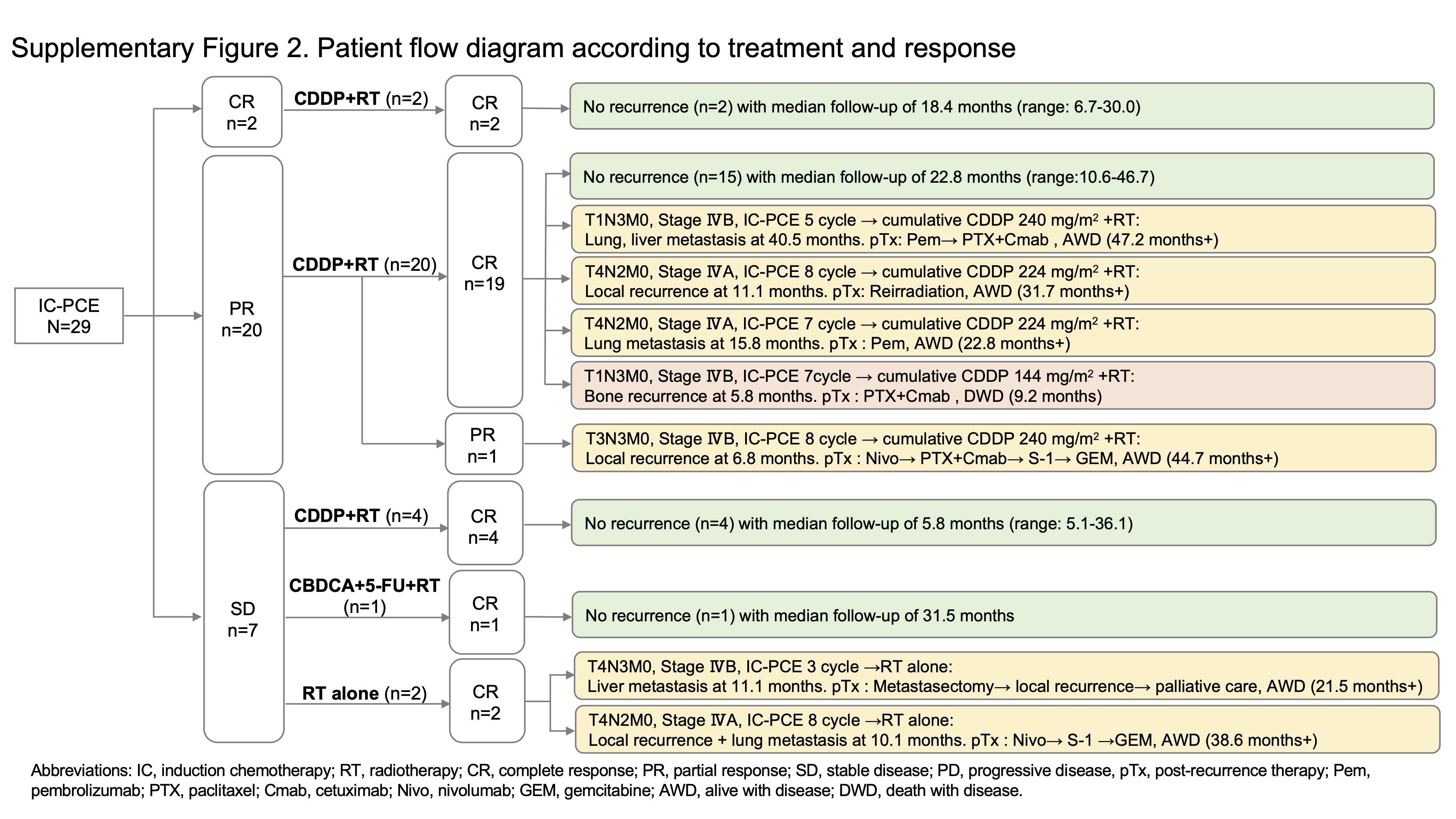

Supplement: Supplementary file 2 [file Image_2.jpg]

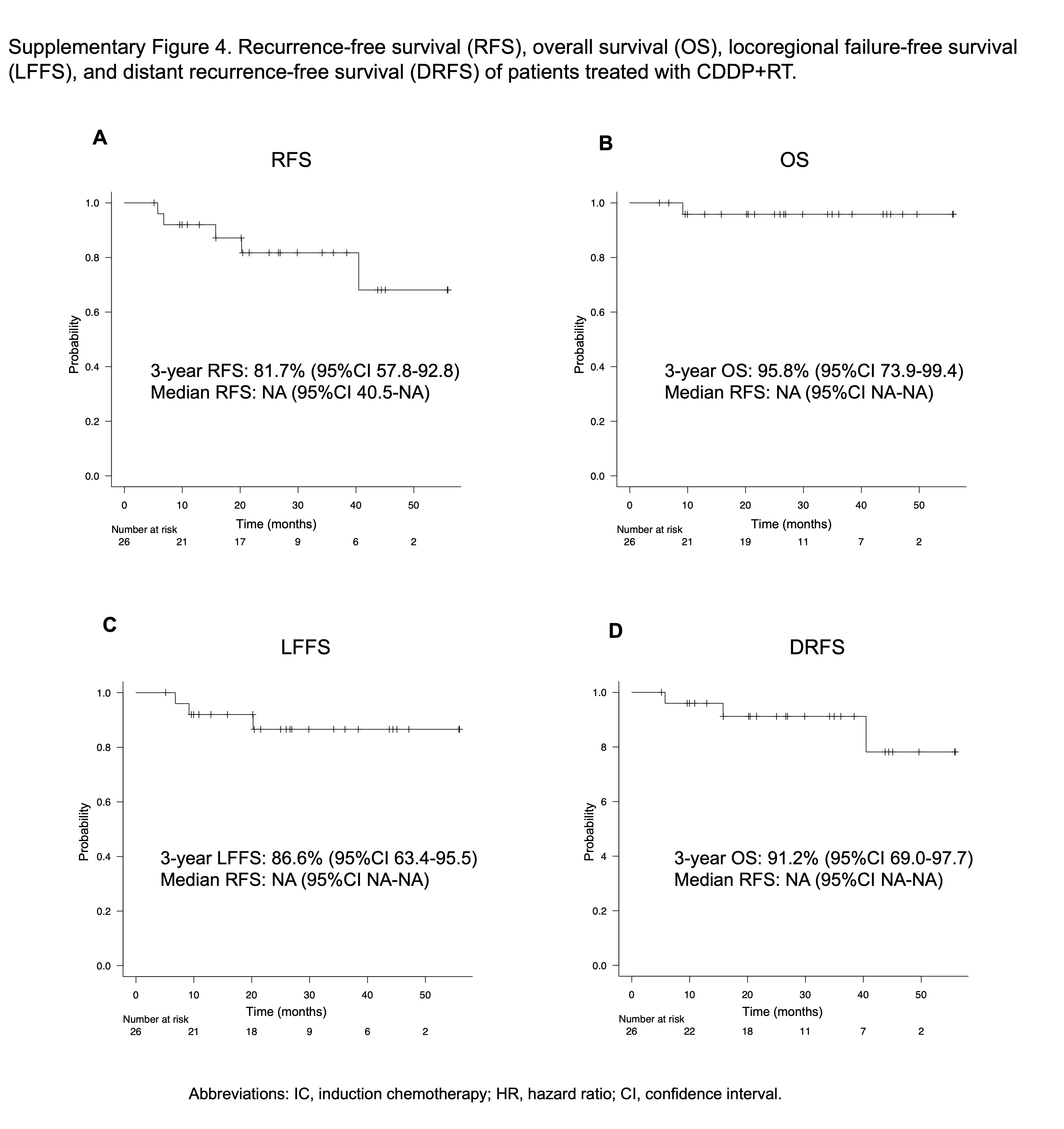

Supplement: Supplementary file 4 [file Image_4.jpeg]
