# Supplementary material for: Induction chemotherapy with paclitaxel, carboplatin and cetuximab for locoregionally advanced nasopharyngeal carcinoma: A single-center, retrospective study
Source: Front Oncol. 2022 Aug 11;12:951387. doi: 10.3389/fonc.2022.951387 (PMC9402945; doi:10.3389/fonc.2022.951387)
Supplement: Supplementary file 5 [file Table_1.docx]

**Supplementary Table 1. Status of administered IC-PCE**

| No. of cycles administered | No. of patients (%) | Reasons for discontinuation of IC |
| --- | --- | --- |
| 1 | 1 (3.4) | No apparent relief of tumor-related symptom^†^ observed |
| 2 | 1 (3.4) | Acute exacerbation of COPD |
| 3 | 1 (3.4) | No apparent relief of tumor-related symptom^†^ observed |
| 4 | 1 (3.4) | Febrile neutropenia |
| 5 | 3 (10.3) | Thromboembolic event (n=1)  No apparent tumor shrinkage observed (n=2) |
| 6 | 1 (3.4) | ALT elevation |
| 7 | 5 (17.2) | Neutropenia (n=2)  Rash (n=1)  Soft tissue infection (n=2) |
| 8 | 16 (55.2) | - |
| Median no. of cycles administered (range) | 8 (1-8) | |

Abbreviations: AE, Adverse event; COPD, chronic obstructive pulmonary disease. ^†^Dysphagia from cranial nerve palsy due to the primary tumor.
