# Supplementary material for: Induction chemotherapy with paclitaxel, carboplatin and cetuximab for locoregionally advanced nasopharyngeal carcinoma: A single-center, retrospective study
Source: Front Oncol. 2022 Aug 11;12:951387. doi: 10.3389/fonc.2022.951387 (PMC9402945; doi:10.3389/fonc.2022.951387)
Supplement: Supplementary file 6 [file Table_2.docx]

**Supplementary Table 2. Compliance with IC-PCE**

|  | PTX | CBDCA | Cmab |
| --- | --- | --- | --- |
| ﻿**No. of administrations** | | | |
| Median | 7 (1-8) | 7 (1-8) | 8 (1-8) |
| ﻿**Cumulative dose**^†^ | | | |
| Median (range) | 560 (80-640) | 10.5 (1.5-12) | 2150 (400-2150) |
| Mean | 486.6 | 9.43 | 1805.2 |
| ﻿**RDI**^‡^ (%) | | | |
| Median (range) | 87.5 (12.5-100) | 87.5 (12.5-100) | 100 (18.6-100) |
| Mean | 78.5 | 76.0 | 84.0 |

Abbreviations: PTX, paclitaxel; CBDCA, carboplatin; Cmab, cetuximab, RDI: relative dose-intensity. ^†^Cumulative dose delivered during the induction phase. Units of measurement are as follows: PTX, mg/m^2^; CBDCA, AUC (area under the curve), Cmab, mg/m^2^. ^‡^Ratio of actual dose intensity to planned dose intensity, accounting for treatment delays and dose reductions.
