# Supplementary material for: Induction chemotherapy with paclitaxel, carboplatin and cetuximab for locoregionally advanced nasopharyngeal carcinoma: A single-center, retrospective study
Source: Front Oncol. 2022 Aug 11;12:951387. doi: 10.3389/fonc.2022.951387 (PMC9402945; doi:10.3389/fonc.2022.951387)
Supplement: Supplementary file 7 [file Table_3.docx]

**Supplementary Table 3. Compliance with CDDP+RT**

|  | N=26^†^ |
| --- | --- |
| **Cisplatin (CDDP)** | |
| Median cycle of CDDP administration (range) | 3 (1-3) |
| No. of patients completing three cycles (%) | 23 (88.5) |
| Median RDI [%] (range) | 100 (33.3 - 100) |
| Mean cumulative CDDP dose [mg/m^2^] ± SD (range) | 213 ± 56.9 (80 – 240) |
| No. of patients with cumulative dose ≥200 mg/m^2^ (%) | 23 (88.5) |
| **Radiotherapy (RT)** | |
| Schedule  70.0 Gy/35 fractions  69.96 Gy/33 fractions | 10 (38.5)  16 (61.5) |
| Omission of RT  No (%)  Yes (%) | 26 (100)  0 (0) |
| ﻿ Total days of omission of RT [days] | 0 |
| Median length of RT [days] (range) | 48 (44 - 51) |
| **Rate of CRT completion**^‡^ (%) (95% CI) | 88.5 (70.2 – 96.8) |

Abbreviations: CRT, chemoradiotherapy; SD, standard deviation; CI, confidence interval. ^†^Among patients receiving CDDP+RT as local therapy following induction chemotherapy. ^‡^Proportion of CRT completion was defined by (a) completion of planned CDDP relative to dose intensity (RDI) ≥80% equivalent to a dose of 200mg/m^2^; and (b) completion of radiotherapy within two weeks after the planned completion date.
