# Supplementary material for: Induction chemotherapy with paclitaxel, carboplatin and cetuximab for locoregionally advanced nasopharyngeal carcinoma: A single-center, retrospective study
Source: Front Oncol. 2022 Aug 11;12:951387. doi: 10.3389/fonc.2022.951387 (PMC9402945; doi:10.3389/fonc.2022.951387)
Supplement: Supplementary file 8 [file Table_4.docx]

**Supplementary Table 4. Comparison of patient characteristics with other studies**

|  | No. of patients (%) | | |
| --- | --- | --- | --- |
|  | IC-PCE [N=29]  (Present study) | IC-TPF →CDDP+RT [N=241]  (Lancet Oncol 2016^4^) | IC-GP →CDDP+RT [N=242]  (N Engl J Med 2019^5^) |
| **Age, years** | | | |
| Median(range) | 59 (24-75) | 43 (36-49) | 46 (18-64) |
| **Gender** | | | |
| Male/female | 22 (75.9)/7 (24.1) | 193 (80)/48 (20) | 182 (75.2)/60 (24.8) |
| **PS** | | | |
| ECOG 0/1 | 25 (86.2)/4 (13.8) | N.R | N.R |
| Karnofsky  90-100/70-80 | - | 217 (90)/24 (10) | 199 (82.2)/43 (17.8) |
| **T category** | | | |
| 1 | 7 (24.1) | 15 (6) | 3 (1.3) |
| 2 | 2 (6.9) | 27 (11) | 16 (6.7) |
| 3 | 4 (13.8) | 112 (47) | 115 (47.5) |
| 4 | 16 (55.2) | 87 (36) | 109 (45.0) |
| **N category** | | | |
| 1 | 8 (27.6) | 97 (40) | 114 (47.1) |
| 2 | 8 (27.6) | 105 (44) | 101 (41.7) |
| 3a | 0 (0) | 13 (5) | 12 (5.5) |
| 3b | 13 (44.8) | 26 (11) | 15 (6.2) |
| **M category** | | | |
| 0 | 29 (100) | 241 (100) | 242 (100) |
| 1 | 0 (0) | 0 (0) | 0 (0) |
| **cStage**^†^ | | | |
| Ⅲ | 7 (24.1) | 129 (54) | 111 (45.9) |
| ⅣA | 10 (34.5) | 73 (30) | 104 (43.0) |
| ⅣB | 13 (44.8) | 39 (16) | 27 (11.2) |

Abbreviations: ECOG PS, Eastern Cooperative Oncology Group Performance Status, IC, induction chemotherapy; TPF, docetaxel+cisplatin+cetuximab; GP, gemcitabine+ cisplatin; CDDP, cisplatin; RT, radiotherapy; N.R, not reported. ^†^AJCC 7th
