# Supplementary material for: Induction chemotherapy with paclitaxel, carboplatin and cetuximab for locoregionally advanced nasopharyngeal carcinoma: A single-center, retrospective study
Source: Front Oncol. 2022 Aug 11;12:951387. doi: 10.3389/fonc.2022.951387 (PMC9402945; doi:10.3389/fonc.2022.951387)
Supplement: Supplementary file 9 [file Table_5.docx]

**Supplementary Table 5. Comparison of Grade 3/4 adverse events with other studies**

|  | No. of patients (%) | | |
| --- | --- | --- | --- |
|  | IC-PCE [N=29]  (Present study) | IC-TPF→CDDP+RT[N=241]  (Lancet Oncol 2016^4^) | IC-GP →CDDP+RT[N=242]  (N Engl J Med 2019^5^) |
| **During IC** | | | |
| Leukopenia | 3 (10.3) | 65 (27.2) | 26 (10.9) |
| Neutropenia | 7 (24.1) | 84 (35.1) | 49 (20.5) |
| Febrile neutropenia | 1 (3.4) | 4 (1.7) | 0 (0) |
| Anemia | 0 (0) | 1 (0.4) | 4 (1.7) |
| Thrombocytopenia | 0 (0) | 0 (0) | 13 (5.4) |
| Allergic reaction | 0 (0) | 2 (0.8) | 1 (0.4) |
| Hepatoxicity | 1 (3.4) | 6 (2.5) | 5 (2.1) |
| Renal toxicity | 0 (0) | 0 (0) | 3 (1.3) |
| Nausea | 0 (0) | 10 (4.2) | 22 (9.2) |
| Vomiting | 0 (0) | 8 (3.4) | 26 (10.8) |
| Mucositis | 0 (0) | 15 (6.2) | 2 (0.8) |
| Diarrhea | 0 (0) | 19 (8.0) | 1 (0.4) |
| Rash | 2 (6.9) | N.R | N.R |
| **Treatment-related death** | 0 (0) | 1 (0.4) | 0 (0) |
| **Total with ≥Grade 3 toxicity** | 10 (34.5) | 102 (42.3) | 93 (38.9) |
| **During CRT** | | | |
| Leukopenia | 5 (17.2) | 65 (27.2) | 47 (19.7) |
| Neutropenia | 5 (17.2) | 54 (22.6) | 28 (11.7) |
| Febrile neutropenia | 1 (3.4) | 3 (1.2) | 1 (0.4) |
| Anemia | 0 (0) | 4 (1.7) | 19 (7.9) |
| Thrombocytopenia | 0 (0) | 6 (2.5) | 17 (7.1) |
| Hepatoxicity | 0 (0) | 1 (0.4) | 1 (0.4) |
| Renal toxicity | 0 (0) | 0 (0) | 3 (1.3) |
| Nausea | 1 (3.4) | 49 (20.5) | 43 (18.0) |
| Vomiting | 0 (0) | 53 (22.2) | 42 (17.5) |
| Mucositis | 6 (20.7) | 92 (38.5) | 67 (28.0) |
| Dry mouth | 0 (0) | 13 (5.4) | 12 (5.0) |
| Diarrhea | 0 (0) | 0 (0) | 5 (2.1) |
| Dermatitis | 0 (0) | 9 (3.7) | 5 (2.1) |
| **Treatment-related death** | 0 (0) | 0 (0) | 0 (0) |
| **Total with ≥Grade 3 toxicity** | 13 (44.8) | 142 (59.4) | 156 (65.3) |

Abbreviations: IC, induction chemotherapy; TPF, docetaxel+cisplatin+cetuximab; GP, gemcitabine+cisplatin; CDDP, cisplatin; RT, radiotherapy; N.R, not reported.
